# How to Gargle the Naso-Pharynx

**Published:** 1880-02

**Authors:** 


### HOW TO GARGLE THE NASO-PHARYNX.

WHEN the gargle is designed to reach the naso-pharynx, Dr. Löwenburg recommends the following method:

The patient inclines the head horizontally backward, and performs movements which we may call "quasi-deglutition," not including the last portion of this physiological action, definite swallowing. The liquid is passed much higher behind the soft palate than the ordinary method of gargling will permit; some persons succeed so well in this manœuvre that they are able to reject by the nose the liquid which has been received by the mouth. Moreover, these rapid muscular contractions completely detach the abnormal secretions, which can then be easily expelled, and the greatest possible relief is thus given to the patient.

